# *Siamothrips balteus*, a new species of *Scirtothrips* genus-group from China (Thysanoptera, Thripidae)

**DOI:** 10.3897/zookeys.637.10910

**Published:** 2016-12-02

**Authors:** Zhaohong Wang, Xiaoli Tong

**Affiliations:** 1 Department of Entomology, College of Agriculture, South China Agricultural University, Guangzhou 510642, China

**Keywords:** China, new species, Siamothrips, thrips, Thripinae

## Abstract

The third species of the genus *Siamothrips* Okajima, *Siamothrips balteus* **sp. n.**, is described from China. The new species is characterised by the abdominal tergite II uniformly brown, III–VII with a brown area medially but pale on lateral thirds, tergite VIII smooth medially, tergite X with 3–4 rows of microtrichia medially, and abdominal sternite VII with one pair of discal setae laterally. A key to the three species has been constructed and is presented here.

## Introduction

*Siamothrips* is a small genus that was erected by Okajima (1990) based on a single species, *Siamothrips argus* Okajima, from Thailand. Subsequently, Ng and Mound (2015) described a second species in the genus, *Siamothrips initium* Ng & Mound from Malaysia, and a third species is described here from southern China. Although the generic relationships of the genus are not clear, Okajima (1990) suggested placing it in the tribe Sericothripini, but more recently Masumoto and Okajima (2007) suggested *Siamothrips* might be a member of the *Scirtothrips* genus-group.

## Materials and methods

The thrips were collected by beating vegetation over a white plastic tray using a stick, and then sorted and preserved in 90% alcohol. Examined specimens were mounted with Canada balsam using the method outlined by Zhang et al. (2006). Details of the morphological structures were examined with a ZEISS Imager A1 microscope, the photos were taken by the Photometrics CoolSNAP camera, and the figures were subsequently processed with Adobe Photoshop CS6. All type specimens are deposited in the Insect Collection, South China Agricultural University (SCAU).

## Taxonomy

### Key to species of *Siamothrips* (female)

**Table:** 

1	Body and wings uniformly pale; median and submedian setae on mesoscutum not arranged in a transverse line; meso- and metascutum sculpture without inner markings [Thailand]	***Siamothrips argus***
–	Body and wings bicoloured, pale to brown; median and submedian setae on mesoscutum arranged in a transverse line; sculpture on meso- and metascutum bearing inner markings	**2**
2	All abdominal tergites pale; tergite VIII with rows of microtrichia extending across segment on anterior half; tergite X smooth medially; abdominal sternite VII without any discal setae and median pair setae arising in front of posterior margin [Malaysia]	***Siamothrips initium***
–	Abdominal tergite I pale, II uniformly brown, III–VII with brown area medially but pale on lateral thirds; tergite VIII smooth medially; tergite X with 3–4 rows of microtrichia medially; abdominal sternite VII with one pair of discal setae laterally and median pair setae situated at posterior margin [China]	***Siamothrips balteus* sp. n.**

### Siamothrips balteus sp. n.

Taxon classificationAnimaliaThysanopteraThripidae

http://zoobank.org/E3D023EA-0949-4C85-94A7-FC7D6466AA67

Figs 1–10

#### Material examined.

**Holotype.** 1 female: **CHINA**, Jiangxi province, Jing’an County, Sanzhualun National Forest Park, Luojiaping (29°01'33"N, 115°17'32"E, alt. 630m), collected from young leaves of *Loropetalum chinense* (Hamamelidaceae), 17.viii.2016, leg. Zhaohong Wang.

**Paratypes.** 18 females, same data as holotype.

#### Diagnosis.

Body bicoloured, pale to brown; fore wing pale except brown submedianly; abdominal tergite II uniformly brown in contrast to largely pale colouration of the other tergites. Antennal segments III and IV with sense cones forked. Median and submedian setae on mesoscutum arranged in a transverse line; sculpture on meso- and metascutum bearing inner markings. Abdominal tergite VIII smooth medially; tergite X with 3–4 rows of microtrichia medially; abdominal sternite VII with one pair of discal setae laterally and median pair setae situated at posterior margin.

#### Description.

**Figures 1–10.**
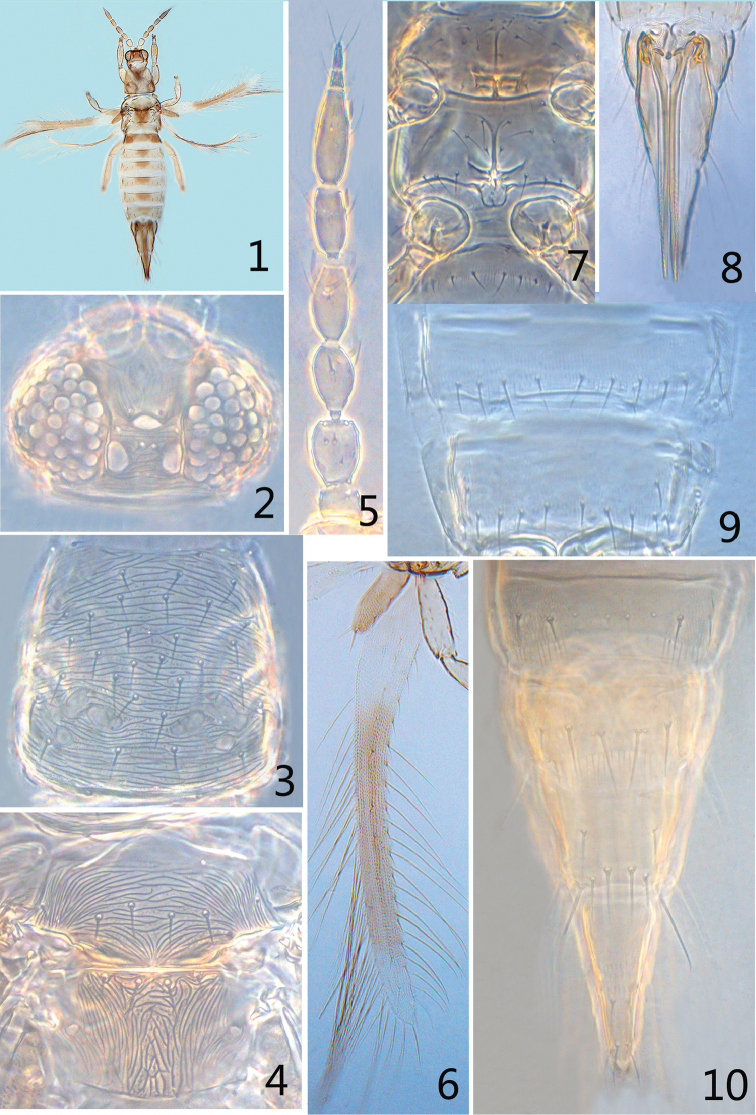
*Siamothrips balteus* sp. n. **1** female habitus **2** head **3** pronotum **4** meso- and metanotum **5** antenna **6** fore wing **7** meso- and metasternum 8 ovipsitor **9** abdominal sternites VI–VII **10** abdominal tergites VII–X.

**Female** (*macropterous*) (Fig. 1): Body pale to brown, anterior 3/4 margin of head yellowish brown; antennal segments I–II pale, III light brown, IV–VIII uniformly brown; pronotum uniformly light yellowish brown; posterior half of mesonotum and metanotum brown; all legs pale but tarsi slightly darker at extreme apex; fore wing brown except pale at basal 1/3 and apical 1/7, clavus brown; abdominal tergite I pale, II uniformly brown, III–VII with brown area medially but pale lateral thirds, and antecostal ridges darker medially, VIII–X uniformly yellowish brown; abdominal sternites pale including antecostal ridges.

*Head* approximately twice as wide as long, widest across eyes, slightly projecting in front of compound eyes; dorsal surface of head including ocellar triangle sculptured with transverse anastomosing striae, but the frons with longitudinal striae (Fig. 2); eyes bulging and pilose without pigmented ommatidia; cheeks very short and almost parallel; three pairs of ocellar setae present, setal pairs I and III subequal in length, pair II longest; pair I situated in front of ocelli, pair II situated on margin or outside of ocellar triangle near eyes, pair III arising on tangent between anterior margins of hind ocelli (Fig. 2); two pairs of postocular setae; mouth-cone long but never extending beyond posterior margin of pronotum; maxillary palps 3-segmented, terminal segment long, slightly shorter than the combined length of other two segments. Antennae 8-segmented (Fig. 5), segment I without dorsal apical setae, II without campaniform sensilla, with four rows of microtrichia dorsally; forked sensoria on III–IV, not reaching more than one-third the length of succeeding segment, V–VI each with an small outer sense cone; III–VI with about four rows of microtrichia on both dorsal and ventral surfaces.

*Pronotum* (Fig. 3) trapezoidal with approximately 35–40 fine setae including marginal setae, without long posteroangular setae; dorsal surface sculptured with distinctly transverse anastomosing striae but on posterior half, the striae are irregular medially. Mesonotum (Fig. 4) with irregular transverse anastomosing striae bearing inner granules, without campaniform sensilla; median and submedian setae arranging in a transverse line. Metanotum (Fig. 4) sculpture irregular longitudinal reticulate medially with inner markings, lateral area with longitudinal lines bearing feeble inner granules, without campaniform sensilla; median setae usually situated near anterior margin (sometimes at anterior margin), submedian setae situated at anterior margin; median setae slightly shorter than submedian setae. Meso- and metasternum each with approximately 20 long fine setae, meso- and metafurcae with spinula (Fig. 7). Fore wing (Fig. 6) first vein with 11–12 setae, second vein without setae, clavus with three veinal and one discal setae; posteromarginal fringe cilia weakly wavy. Tarsi 2-segmented.

*Abdominal tergites* II–VII with closely spaced rows of ciliate microtrichia on lateral thirds, S1 setae small, slightly longer than the distance between their bases, but S1 setae on tergites VIII–IX well developed and long, approximately twice as long as distance between their bases; tergites II–VIII smooth medially without any rows of microtrichia, tergite VIII with complete posteromarginal comb (Fig. 10); tergite IX without campaniform sensilla or microtrichia, but tergite X with 3–4 rows of microtrichia medially (Fig. 10). Abdominal sternites II–VII with rows of ciliate microtrichia across median area, at least on posterior halves, posterior margin with fringe of microtrichia; segment II with three pairs of long posteromarginal setae, III with four pairs of long posteromarginal setae; IV–VII with five pairs of long posteromarginal setae, all primary setae of sternites situated at posterior margins; sternite VII with one pair of discal setae laterally (Fig. 9). Ovipsitor (Fig. 8) straight and elongate, slightly longer than twice the length of pronotum.

Male unknown.

*Measurements* (holotype female in microns). Distended body length 940. Head, dorsal length 50, ventral length to mouth cone tip 175, width across compound eyes 100; ocellar setae II 16; ocellar setae III 10; postocular setae I 9. Eye length 45. Pronotum length 110, maximum width 125. Metascutal median setae 17, submedian setae 18. Fore wing length 430. Length of median setae on abdominal tergite II–VII 5–10, on tergite VIII–IX 35–45. Antennal segments I–VIII length (width) as follows: 17(20), 26(23), 35(18), 33(18), 34(15), 42(14), 8(6), 12(4). Ovipositor length 240.

#### Etymology.

The specific epithet is from the Latin *balteus*, meaning “belt or waistband,” in reference to the abdominal tergite II being entirely brown in contrast to largely pale colouration of the other tergites.

#### Distribution.

China (Jiangxi).

#### Remarks.

This new species is most similar to *Siamothrips initium* Ng & Mound, 2015 from Malaysia; however, it can be distinguished from these two species by the key above.

## Supplementary Material

XML Treatment for Siamothrips balteus
